# Effect of Recycled Fine Aggregates on the Mechanical and Drying Shrinkage Properties of Alkali-Activated Recycled Concrete

**DOI:** 10.3390/ma17092102

**Published:** 2024-04-29

**Authors:** Ling Luo, Wu Yao, Gang Liao

**Affiliations:** 1 Key Laboratory of Advanced Civil Engineering Materials of Ministry of Education, School of Materials Science and Engineering, Tongji University, Shanghai 201804, China; 2011591@tongji.edu.cn (L.L.); gangliao@tongji.edu.cn (G.L.); 2 College of Civil Engineering and Architecture, Xinjiang University, Urumqi 830017, China

**Keywords:** alkali-activated recycled concrete, mechanical performance, drying shrinkage, microstructure

## Abstract

In this paper, the workability, mechanical, ion leaching, and drying shrinkage properties of alkali-activated concrete with recycled coarse and fine aggregates were studied, and the pore structure and micro-morphology of different alkali-activated recycled aggregate concretes (AARACs) were characterized by using the mercury intrusion method and scanning electron microscopy, respectively. The experimental results showed that with the increase in the replacement rate of the recycled fine aggregate (RFA), the flowability showed a decreasing trend. Adding a certain amount of RFA improves the mechanical properties of the AARAC. The compressive strength at a curing age of 28 days was 65.3 MPa with 70 wt% RFA replacement. When the replacement rate of the RFA was 100 wt%, the maximum splitting tensile strength (4.5 MPa) was obtained at a curing age of 7 days. However, the addition of the RFA had little effect on the flexural strength of the AARAC. As an extension of the curing age, the splitting tensile strength, flexural strength, tension-to-compression ratio, and flexure-to-compression ratio all showed an increasing trend at first and then a decreasing trend. At a curing age of 7 days, the tension-to-compression ratio and flexure-to-compression ratio were both high (except for those of R100), indicating that the ductility and toughness of the specimen were improved. The addition of the RFA increased the drying shrinkage of the AARAC. At a curing age of 120 days, compared to the specimen without the RFA, the drying shrinkage rate of the specimen with the addition of 70 wt% RFA increased by 34.15%. As the curing age increased, the microstructure of the reaction products became denser, but the proportion of large-diameter pores increased. This study evaluated the application of RFA in AARAC. The experimental results showed that the RFA-based AARAC had acceptable mechanical and durability properties.

## 1. Introduction

Currently, concrete is one of the most extensively used construction materials, and its global average annual consumption is approximately 11 billion tons [1]. The excessive exploitation of natural resources such as sand and gravel has resulted in a scarcity of native resources and soil erosion, creating severe ecological and environmental problems [1,2]. Consequently, it is crucial and meaningful to find an environmentally friendly and stable composite material to replace some concrete materials [3].

In China, over 1.8 billion tons of construction and demolition waste (C&DW) are produced annually, 40% of which is waste concrete [4]. At present, the main disposal method of C&DW in China is stacking it in landfills, which is harmful to the ecological environment [5,6]. Moreover, it is estimated that the carbon dioxide emissions from the cement industry account for approximately 57% of global emissions [7,8,9]. The generation of the large volume of construction waste and the emission of carbon dioxide gas from cement manufacturing have a devastating impact on the environment. Therefore, in order to address the above-mentioned environmental and resource issues, some new types of concrete, such as alkali-activated concrete and recycled concrete, have been proposed.

Alkali-activated materials (AAMs) are typically made from ground granulated blast-furnace slag (GGBS), fly ash (FA), calcined clay, and other aluminate precursors [10,11,12]. Currently, numerous studies have successfully utilized solid waste materials to prepare geopolymer materials with various properties [13,14]. Regarding slag/fly ash-based geopolymers, both domestic and international scholars have extensively researched their preparation processes and properties [15,16]. Alkali-activated slag (AAS) concrete has excellent performance in terms of its early strength and corrosion resistance [17,18,19], but it also has some drawbacks, such as its large shrinkage and low toughness values [20,21], which limit its widespread application. Recycled aggregate (RA) is produced from waste concrete through crushing, shaping, screening, and other processes. The main difference between RA and natural aggregate is that the former has some old mortar adhering to its surface, and the aggregate particles contain a large number of microcracks formed during the crushing process of the waste concrete. The attached mortar and internal microcracks result in the recycled aggregate’s low apparent density, high water absorption, low strength, and rough surface [22,23,24], which leads to significant differences in performance between recycled aggregate concrete and natural aggregate concrete [25]. The density and absorption capacity of RA are influenced by the attached mortar [26,27]. When natural coarse aggregate was fully replaced by recycled coarse aggregate (RCA), the compressive strength was reduced by up to 30% in AAS concrete [28,29,30]. Aguilar et al. [31] reported that the porous and cracked structure of RA in AAS concrete resulted in a reduction in its compressive strength. The extra water used in RA reduced the dissolution rate of the silica-aluminate precursor of the alkali-activated matrix [32]. Sata et al. [33] showed that the incorporation of RCA generated a weakened interfacial transition zone in AAS concrete. In addition, the water absorption and permeable void volume of AAS concrete increased with an increase in a recycled aggregate admixture [34,35]. Kathirvel and Kaliyaperumal [28] reported that the compressive and flexural strengths of AAS concrete increased slightly, less than 10%, when 50% wt% natural aggregate was replaced with RCA. Cantero et al. [14] studied various replacement levels of recycled structure and demolition wastes in the production of structural concrete. Sun et al. developed self-compacting concrete based on RA, and the replacement levels of the RA ranged from 0 wt% to 100 wt% compared to other aggregates [4,36,37,38,39]. Therefore, the literature has shown that many structural concretes made of RA have good engineering performance. Aguilar et al. [31] reported that the high porosity and microcracks of RA led to a decrease in the compressive strength of AAS concrete. The extra water absorbed by RA reduced the dissolution rate of the aluminosilicate precursors in an alkali-activated matrix [32]. Kathirvel and Kaliyaperumal [28] reported that when the replacement rate of RCA to natural gravel was 50 wt%, the compressive and flexural strength of AAS concrete increased by less than 10% [36,37,38,40]. It has been reported that RFA in concrete may decrease its compressive strength by 21% to 60% and adversely affect its durability. Unlike RCA, RFA has more cement paste on its surface, and the attached cement pastes increase the porosity of the concrete. In addition, due to the complex composition of RFA, the replacement rate is highly restricted, and it is recommended that the replacement rate of RFA in concrete should not exceed 15% [40] or 20% [41]. In practice, the use of RFA in the production of concrete is highly restricted or even banned in some countries [42,43,44].

Based on this context, this study firstly attempted to fully replace natural coarse aggregates with RCAs for the manufacturing of alkali-activated recycled concrete. Then, the effects of the replacement rate of RFA on the fluidity, compressive strength, flexural strength, splitting tensile strength, and dry shrinkage of alkali-activated recycled concrete were investigated. In addition, the pore structure of alkali-activated recycled concrete was characterized by the mercury intrusion method. This work was a preliminary study on the preparation of alkali-activated recycled concrete using bulk solid wastes, which was expected to provide new insights into the development of green recycled concrete.

## 2. Materials and Methods

### 2.1. Materials

The materials of binder consist of ground granulated blast-furnace slag (GGBS) and fly ash (FA). The GGBS and FA were obtained from Gongyi and Zhengzhou (Henan, China), respectively. Their chemical compositions are listed in Table 1. Using a volumetric flask and kerosene, the particle densities of FA and GGBS measured by the Archimedes method were 2.42 and 2.93 g/cm^3^, respectively. The median particle size (D50) parameters of FA and GGBS were 1.83 μm and 3.19 μm. The alkaline activator with a modulus of 1.8 (molar ratio of SiO_2_ to Na_2_O) was prepared by blending sodium hydroxide pellets (purity ≥96%) and a commercially available sodium silicate solution (8.54 wt% Na_2_O and 27.3 wt% SiO_2_). According to literature [45,46], the proportion of activator solution was 8% Na_2_O (by weight of binder). After stirring, the activator solution was immediately transferred to an “air-tight container”. Prior to its use in concrete, it must be placed at ambient temperature for at least 24 h to reach equilibrium.

**Figure 1:**
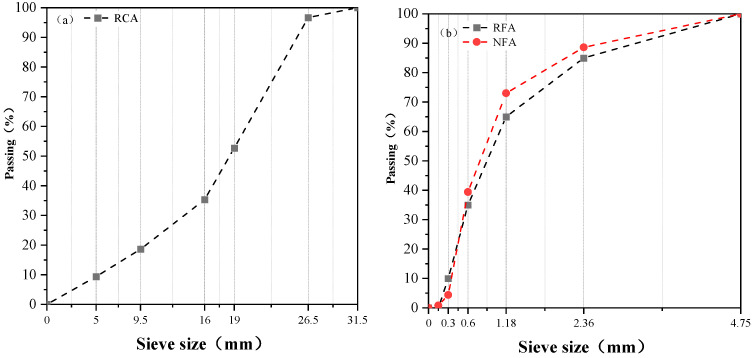
Particle size distribution of different aggregates. (**a**) RCA; (**b**) RFA and NFA.

The recycled coarse aggregate (RCA) used in this study was derived from a new building materials factory in Shanghai. RCAs used in study have two particle size ranges, namely, 5–16.5 mm and 16.5–31.5 mm. The blended RCAs were measured according to national standard GB/T14685-2011 [47]. The recycled fine aggregate (RFA) was taken from a construction waste disposal plant in Shaoxing, Zhejiang Province, and its particle size was less than 4.75 mm. The natural fine aggregate (NFA) was taken from Poyang Lake and had continuous particle grading. In order to ensure the continuity of the particle size distribution of the recycled fine aggregates used in the test, the particles with different particle sizes were initially sieved according to national standard GB/T 14684-2011 [48], and then artificial blending was carried out according to the standard grading ranges in order to ensure the continuous grading of the RFA for the test. According to national standards GB/T 25177-2010 [49] and GB/T 25176-2010 [50], the properties of aggregates were evaluated, and the test results are shown in Table 2. The different particle sizes of aggregates are shown in Figure 1.

Tap water was used for mixing concrete and producing alkaline activator. According to the requirements of JGJ 63-2006 [51], it was ensured that tap water was free from impurities and any harmful substances.

### 2.2. Mixture Proportions

The system was modified with reference to previously published literature [52,53,54] and previous research results. The experiments were carried out with two cementitious materials of GGBS and FA in the ratios of 9:1. Alkali-activated recycled aggregate concrete (AARAC) was prepared using the preferred sodium silicate solution (with a modulus of 1.8 and alkali concentration of 8.0%) as an exciter. RFA was incorporated into the specimens to replace 0 wt%, 30 wt%, 50 wt%, 70 wt%, and 100 wt% NFA. Liquid:binder ratio (mass ratio of alkali solution to cementitious material) was 0.65, and the concrete sand rate was 40%. The mix proportion of AARAC is depicted in Table 3.

**Figure 2:**
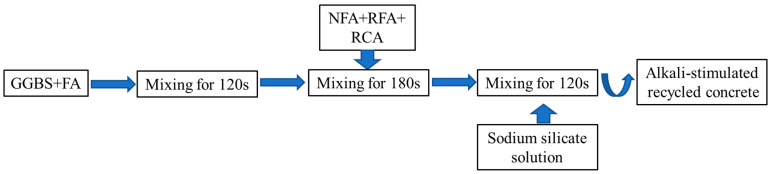
The process of alkali-activated recycled concrete preparation.

The preparation process of alkali-activated recycled concrete is shown in Figure 2. The specimens were prepared according to national standard GB/T 50080-2019 [55]. After casting, the surface of the casting molds was covered with plastic film to prevent moisture loss. All specimens were demolded after 24 h of casting and then cured in air at a temperature of 20 ± 3 °C, with the surface of specimens covered with plastic sheeting until testing took place.

### 2.3. Test Procedures

#### 2.3.1. Flowability Measurement

The initial flowability of the newly mixed AARAC mixture was tested according to the standard T 0532-2020 [56]. Each specimen was measured at least three times until the error was less than 10%.

#### 2.3.2. Leaching Experiments

Experimental leaching was carried out according to NEN 7341 [57]. The effects of Ca^2+^, Al^3+^, and Si^4+^ on the reaction products of RFA in alkaline solutions were analyzed using 4300DV inductively coupled plasma optical emission spectrometer (ICP-OES).

The artificially prepared RFA was dried and divided into 10 portions of 10 g. Volumes of 200 mL NaOH solutions with molar concentrations of 0 and 5 mol/L were prepared and labeled as A and B. The two NaOH solutions were divided into five portions of 40 mL each, for a total of 10 portions, which were placed in polyethylene (HDPE) bottles with a capacity of 100 mL. The weighed 10 portions of RFA were sequentially placed into the polyethylene bottles, shaken well, and then sealed in a water bath at a constant temperature of 25 °C. Placement times were 10 min, 3 h, 12 h, 24 h, and 48 h to form 10 test protocols. Table 4 shows the filtered samples placed under constant temperature until the specified soaking time. The filtrate was sequentially diluted by 1 mL to meet the concentration requirement (<40 mg·L^−1^) of the ICP-OES instrument. In this experiment, the samples were first diluted 1000-fold with deionized water and then 5-fold with 2% HNO_3_, which allowed for the testing of their ion concentrations.

The leaching data are presented as concentrations of the individual elements in the leachates (mg/g). The leaching rate is calculated as follows:

(1) $$R_{i}=\frac{t\cdot C\cdot V}{M}$$

where R_i_ is the leaching rate (mg/g), M is the mass of RFA used (g), t is the number of dilutions, C is the leaching concentration (mg/L), and V is the volume of eluent (L). The rate of increase in the leaching rate of alkali-activated reactive ions relative to that of non-alkali-activated ions was calculated according to Equation (2) for different leaching times.

(2) $$Vr=\frac{Ra-Rw}{Rw}\times 100\%$$

where Vr is the change in leaching rate (%), Ra is the leaching rate (mg/g) in alkali solution (0 and 5.0 mol/L), and Rw is the leaching rate (mg/g) in water (0 mol/L sodium hydroxide solution).

#### 2.3.3. Mechanical Performances

Cubic molds (with the size of 100 mm × 100 mm × 100 mm) and prismatic molds (with the size of 100 mm × 100 mm × 400 mm) were used to prepare the specimens of AARAC for the compressive strength, splitting tensile strength, and flexural strength measurements. According to GB/T50080-2019 [55], immediately after the preparation of concrete, the slump was tested to reflect the workability of AARAC in the freshly mixed state. The fresh mixture was poured into a plastic mold and then compacted by vibration table. The compressive strength and splitting tensile strength were tested using a WAY-3000 electro-hydraulic pressure testing machine, while the flexural strength was tested using an MTS DCS-300 electro-hydraulic pressure testing machine. The testing method was accordance with the “Standard for Testing Mechanical Properties of Ordinary Concrete” (GB/T50081-2019) [58], with test load rates of 0.5 MPa/s, 0.05 MPa/s, and 0.05 MPa/s, respectively.

#### 2.3.4. Drying Shrinkage Measurement

Drying shrinkage was very crucial to the mechanical properties and durability of concrete. The drying shrinkage of AARAC was measured according to DL/T 5150-2001 [59]. Three specimens were prepared for per test. After 24 h of curing, the specimens were removed from the mold and moved into the curing room (with a temperature of 20 ± 2 °C and relative humidity of 80 ± 5%) for 48 h. The initial lengths of the specimens were measured and recorded using a vertical comparator. Then the specimens were moved to a shrinkage chamber (with a temperature of 20 ± 2 °C and a relative humidity of 50 ± 5%), and the length changes of the specimen were recorded 1, 3, 7, 14, 28, 60, 90, and 120 days after initial measurements.

#### 2.3.5. Pore Structure Analysis

The tested samples needed to be crushed into small pieces, and then the collected fragments were immersed in ethanol to terminate the reaction [60,61]. The mercury intrusion porosity (MIP) test was conducted using PoreMaster 33. The pressure range for this test was from 0.23 psi to 33,000 psi, and the aperture range was 1080 μm–5 nm.

#### 2.3.6. Micromorphology Measurement

The instruments used in this test included a scanning electron microscope (SEM, ZEISS Sigma300 Scanning Electron Microscope, Jena, Germany) and an energy-dispersive spectrometer (EDS, Oxford Xplore 30). Before our observations were made, a layer of gold film was sprayed onto the sample.

## 3. Results and Discussion

### 3.1. Flowability Analysis

**Figure 3:**
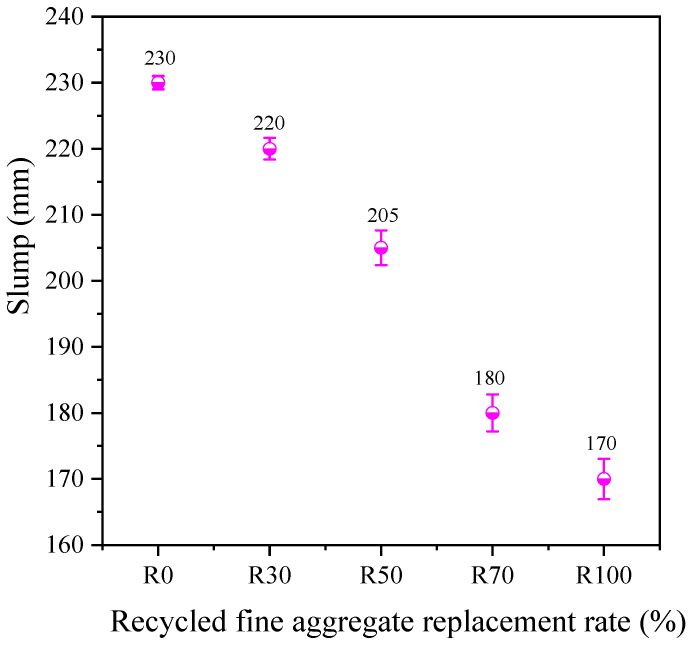
Effect of RFA replacement rate on the flowability of AARAC mixes.

Figure 3 depicts the effect of the RFA replacement rate on the flowability of the AARAC mixture. It can be observed that the slump of the AARAC mixture decreased with the replacement rate of RFA. This was due to the fact that the un-hydrated cement portion of the RFA was covered by the old cement paste, resulting in a higher water absorption rate. As the replacement rate of RFA increased, the excess water was captured by the RFA, consequently increasing the alkali concentration in the mixed paste and accelerating the reaction, thus reducing its fluidity [54]. In addition, the rough surface and numerous angular shapes of the recycled aggregates had an adverse effect on the workability of the AARAC. For example, when the replacement rate of RFA was 100 wt%, the slump of the mixture was only 170 mm, which was 35.3% lower than that of the mixture without the RFA (230 mm).

### 3.2. Mechanical Properties

#### 3.2.1. Compressive Strength

**Figure 4:**
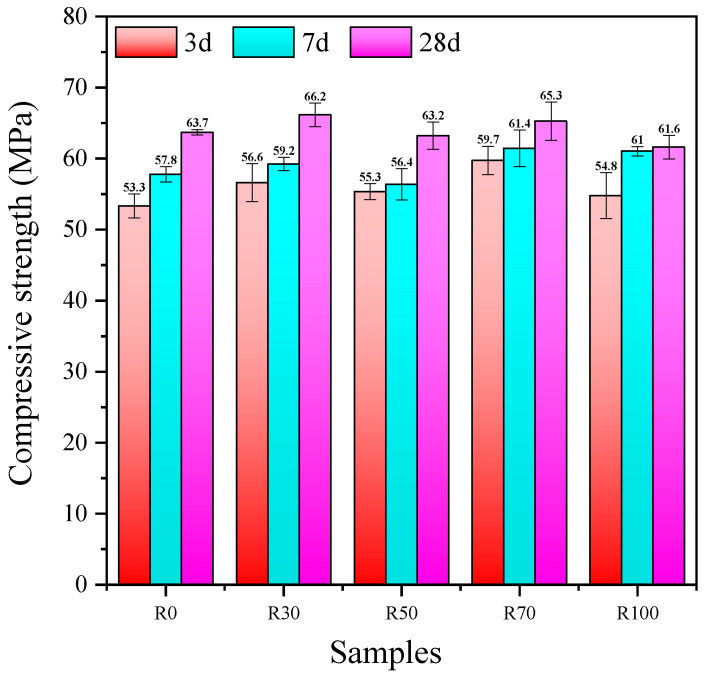
Compressive strength of AARAC mixes upon air curing.

The influence of the replacement rate of the RFA on the compressive strength of the AARAC at different ages is depicted in Figure 4. It can be seen that with the increase in curing age, the compressive strength gradually increased. In the early stage, the compressive strength increased rapidly, while in the later stage, the rate of increase in the compressive strength gradually leveled off. This was mainly because in the alkaline environment (provided by the alkaline activator), the Ca^2+^, Si^2+^, Al^3+^, and other substances in the raw materials (GGBS and FA) dissolved, resulting in the formation of a calcium (sodium) aluminosilicate hydrate gel (C-A-S-H, N-A-S-H), thus leading to a high early compressive strength. The results of this study were in good agreement with those of previous studies [62,63,64]. When the replacement rate of RFA was 100 wt%, the compressive strength at 28 days was only increased by 0.98%, compared with the compressive strength at 7 days. The slow development of strength in the later stage might be related to the damage caused by the shrinkage of the AARAC. In other studies of alkali-activated slag concrete, it has also been reported that drying shrinkage is caused by the formation of an unstable gel phase [65] on the concrete surface and low levels of moisture [66]. The curing age was fixed, and a certain amount of RFA replacement improved the mechanical properties of the AARAC. The specimen with an RFA replacement rate of 70 wt% possessed a high compressive strength (65.3 MPa), indicating the possibility of a high replacement rate of the RFA. Although the incorporation of the RFA increased the number of defects in the mix, the alkali-activated paste was denser and more homogeneous than the ordinary Portland cement slurries, which might reduce the defects caused by RAs [67]. This was similar to the findings of Kathirvel P and Kaliyaperal S R M [28].

#### 3.2.2. Splitting Tensile Strength

**Figure 5:**
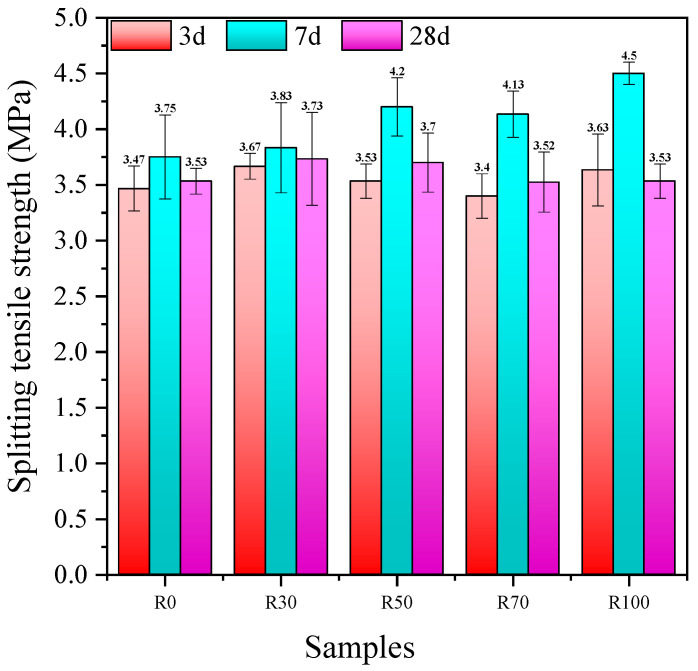
Splitting tensile strength of AARAC mixes upon air curing.

The effect of the replacement rate of the RFA on the splitting tensile strength of the AARAC at different ages is shown in Figure 5. It could be seen that as the curing age increased, the splitting tensile strength initially increased and then decreased. When the replacement rate of the RFA was 100 wt%, the splitting tensile strength at a curing age of 7 days reached a maximum of 4.50 MPa. The splitting tensile strength decreased with the replacement rate of the RFA, and its downward trend was more remarkable than that of the compressive strength. When the replacement rate of the RFA was 100 wt%, compared with the splitting tensile strength at 7 days, the splitting tensile strength at 28 days was decreased by 21.56%. The reason for this might be the addition of the RFA, which increased the internal damage and drying shrinkage of the concrete structure [68,69]. The reason for this might be that the surfaces of the regenerated fine aggregates were covered with the old mortar and contained more micro-cracks, which increased the water absorption of the aggregate, indirectly increasing the concentration of alkali activation. This led to a more intense early reaction, allowing for the rapid formation of a large amount of reaction products in a short period of time and promoting the development of its strength. However, when the reaction progressed to a certain point, with the gradual consumption of the cementitious components and alkali activation, the rate of the reaction gradually slowed down. At the same time, the volume shrinkage of the alkali-activated slag system produces tensile stress, adversely affecting the drying shrinkage of the concrete and exacerbating the internal structural damage of the concrete. Therefore, the late-stage reaction exhibits a strength-reduction phenomenon. This was similar to the findings of Nanayakkara O and Gunasekara C [70].

#### 3.2.3. Flexural Strength

**Figure 6:**
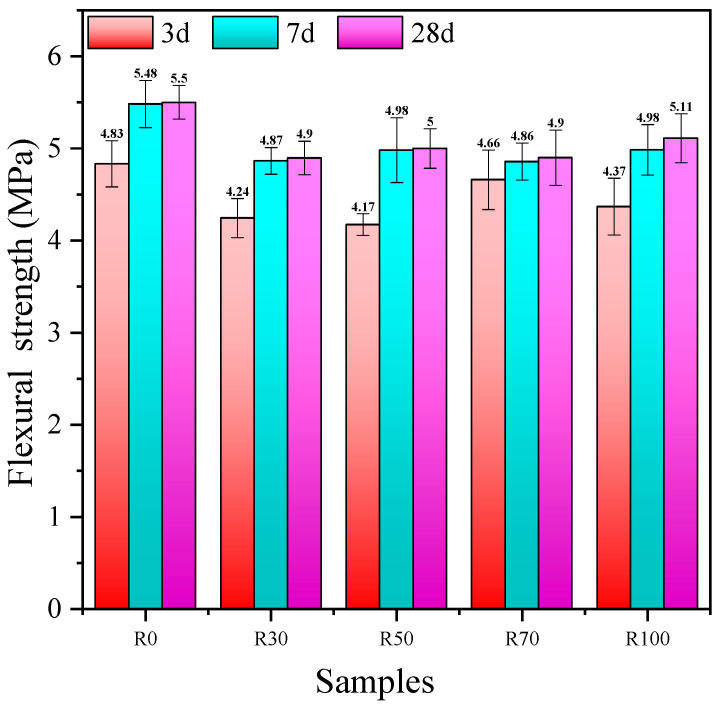
Flexural strength of AARAC upon air curing.

The effect of the replacement rate of the RFA on the flexural strength of the AARAC at different ages is shown in Figure 6. From the experimental results, it can be seen that the flexural strength increases gradually with the increase in the age of curing, but the increase was slower at the later stage and reached the maximum value at 28 days. When the replacement rates of the RFA were 0 and 100 wt%, the flexural strength values at 28 days were 5.5 MPa and 5.11 MPa, respectively, and the flexural strength only decreased by 7.1%, which indicated that the amount of RFA had minimal impact on the flexural strength of the AARAC. The addition of the RFA reduced the flexural strength due to the incorporation of macro-level voids. With the consumption of water, the alkali concentration in the outer layer of the matrix was higher than in the inner layer, and chemicals formed on the surface, resulting in an inhomogeneous matrix. And the damage in the flexural test occurred on the outside, while the damage in the compression test occurred on the inside, which was why the relationship between the flexural strength and compression strength was not obvious. The surface of the RFA was adhered to the old mortar and contained more micro-cracks, which meant the hardened alkali-activated AARAC structure was not dense enough and there were more defects in the interior; thus, the flexural strength growth was slow.

#### 3.2.4. Tension-to-Compression Ratio and Flexure-to-Compression Ratio

**Figure 7:**
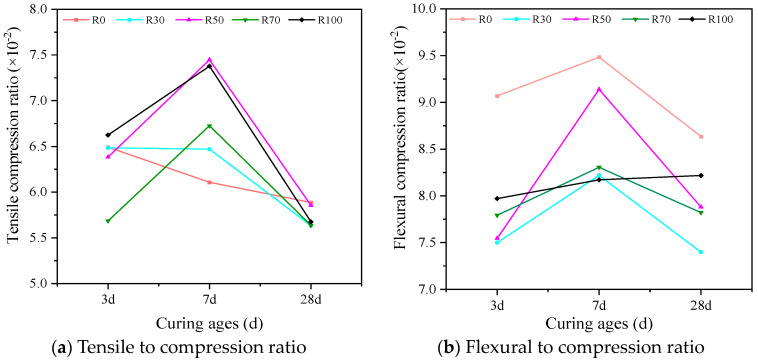
Tensile compression ratio and flexural compression ratio of AARAC with different replacement rates of RFA. (**a**) the ratio of split tensile strength to compressive strength; (**b**) the ratio of flexural strength to compressive strength.

The tension-to-compression ratio (the ratio of split tensile strength to compressive strength) is an indicator of the ductility of concrete; the larger the value, the better the ductility of the concrete. The flexure-to-compression ratio (the ratio of flexural strength to compressive strength) is an indicator of the toughness of concrete; the larger the value, the tougher the concrete [71]. Figure 7 showed the relationship between the replacement rate of the RFA and the tension-to-compression ratio or flexure-to-compression ratio of the AARAC at different curing ages. According to the experimental results, it can be seen that as the curing age increased, the tension-to-compression ratio and the flexure-to-compression ratio both showed a trend of increasing and then decreasing. At a curing age of 7 days, the tension-to-compression ratio and flexure-to-compression ratio were both higher (except R100) than those at other ages, indicating that the specimen had good levels of ductility and toughness. This was mainly due to the generation of a large amount of gel in the early stages of the reaction, which filled the voids between the aggregates, densifying the internal structure and thereby improving the ductility and toughness of the hardened paste. However, in the alkali-activated slag system, due to the rapid reaction rate of the slag, substantial drying shrinkage might occur during the early stages of the reaction, which could lead to the development of numerous micro-cracks in the internal structure. This further exacerbates the damage within the recycled aggregates, resulting in a more pronounced effect on the stiffness, splitting tensile strength, and flexural strength of the recycled aggregate particles [72,73]. In addition, the cracks induced by the alkali activation would ultimately extend to the slurries at a later stage, which would also negatively affect the compressive strength of the material. Therefore, the strength of the AARAC increased slowly and even contracted in the later stages, especially the splitting tensile strength. Pratt PL et al. [74,75] also discussed the advantages and existing problems of alkali-activated slag concrete.

### 3.3. Leaching Processes and Controls

**Figure 8:**
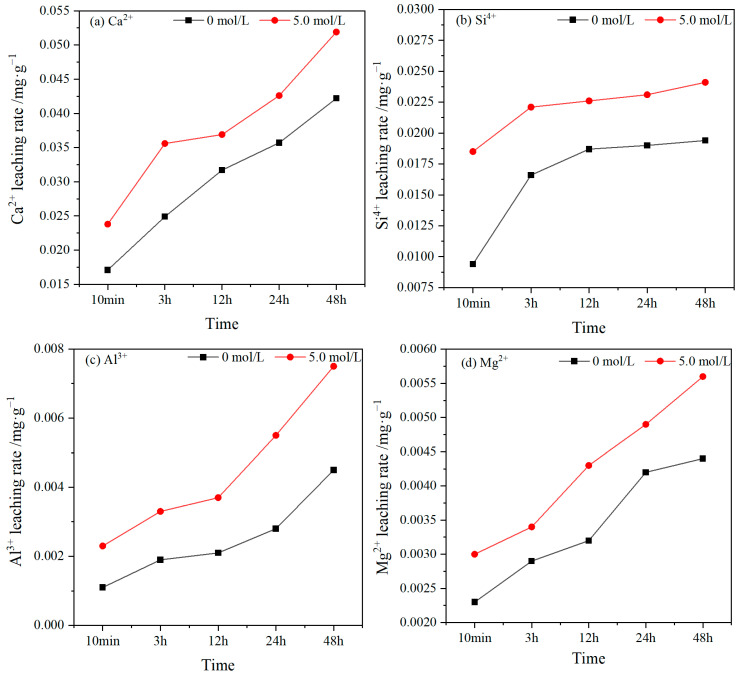
Curves of ion leaching with time.

Figure 8 shows the variation in the leaching rates of Ca^2+^, Si^4+^, Al^3+^, and Mg^2+^ for the RFA when immersed in deionized water and in a NaOH solution with a concentration of 5.0 mol/L over time. From the test results, it can be seen that the amount of ions leaching from the RFA in the alkaline solution was slightly higher than that with the deionized water, but there were also differences in the leaching patterns amongst the four.

The leaching rates of Ca^2+^, Si^4+^, Al^3+^, and Mg^2+^ were different, and the magnitude of the leaching rates was Ca^2+^ > Si^4+^ > Al^3+^ > Mg^2+^. The maximum leaching rate of Ca^2+^ was 0.0589 mg·g^−1^, that of Si^4+^ was 0.0241 mg·g^−1^, that of Al^3+^ was 0.0075 mg·g^−1^, and that of Mg^2+^ was 0.0056 mg·g^−1^ in 48 h. The reason for this is that the content of each component in the RFA is different, and RFA consists of residual sand and residual cement paste. Relative to the deionized water, the NaOH solution increased the leaching rate of Ca^2+^, Si^4+^, Al^3+^, and Mg^2+^ in the RFA at different ages. It can be seen that under the alkali activation, the leaching rates of Ca^2+^, Si^4+^, Al^3+^, and Mg^2+^ were increased to different degrees, with Si^4+^ and Al^3+^ exhibiting the highest leaching rates at 10 min, reaching 96.8% and 109.1%, respectively, Additionally, the leaching rate of Ca^2+^ peaked at 3 h, reaching 43.0%, and Mg^2+^ reached its maximum leaching rate at 12 h, reaching 34.4%. The reason for this was that the un-hydrated cement portion in the RFA was covered by the old cement paste. After the alkali activation, the bond energy of the Al-O bonds was lower than that of the Si-O bonds, which made it easier for Al^3+^ to be solubilized than Si^4+^ [76,77]. Therefore, the leaching rate of Al^3+^ showed the highest increase.

### 3.4. Drying Shrinkage

**Figure 9:**
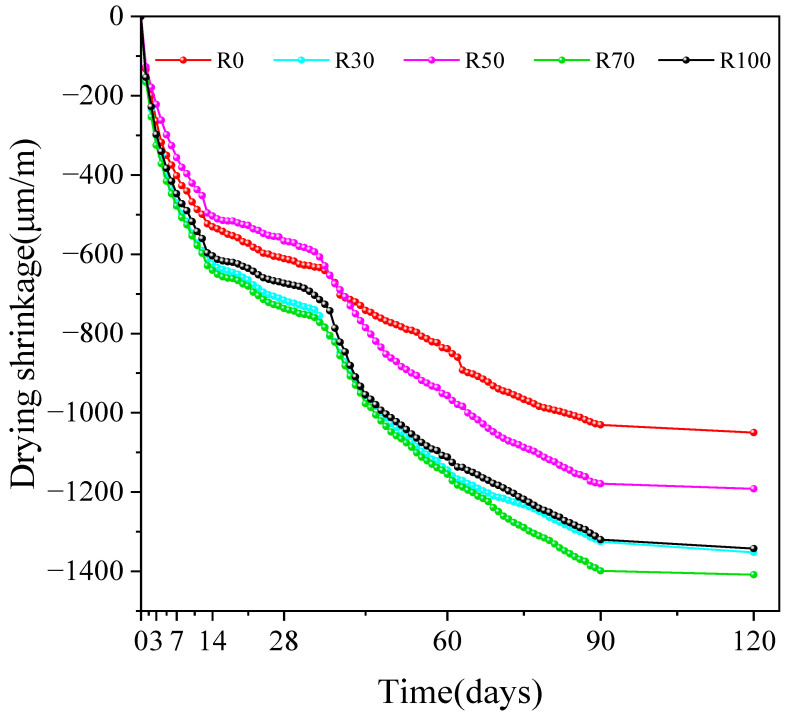
Shrinkage of specimens with different replacement rates of RFA.

Figure 9 shows the relationship between the RFA replacement rate and shrinkage of the AARAC at different curing ages. From the test results, it can be seen that at 120 days, the minimum shrinkage (1049.86 μm/m) was observed in the blank group without the RFA, while the shrinkage of the group with 70 wt% RFA was the highest (1408.37 μm/m), which was 34.15% higher than that of the blank group. The shrinkage rates of the AARAC with replacement rates of 50 wt% and 100 wt% were 1191.73 μm/m and 1342.51 μm/m, respectively. The replacement rate of the RFA had an impact on the drying shrinkage of the AARAC. This was because the water absorption rate of the RFA was higher than that of the NFA, and more internal voids were formed during the hardening process, which led to a relative decrease in the density of the concrete and increase in the drying shrinkage of the concrete.

The amount of early shrinkage was high but slowed down with the increasing curing age. When the alkali activator concentration was 8%, the rate of contraction was significantly slower in all groups between the curing age of 60 and 120 days, and the drying shrinkage of the AARAC was still relatively high. The addition of RFA increased the drying shrinkage of the AARAC. It was also found that the drying shrinkage of the AAS concrete increased continuously with time, but the rate of shrinkage slowed down after a curing age of 28 days [78]. Zhang et al. [79] showed that the drying shrinkage increased by a factor of two when 100% of the coarse aggregate was replaced with recycled aggregate. Kumar S et al. also reported similar conclusions [60,70]. Therefore, while alkali-activated slag recycled concrete has great potential for practical applications in the presence of certain concentrations of alkali activators. It is also necessary to focus on shrinkage reduction in subsequent research.

### 3.5. MIP Analysis

**Figure 10:**
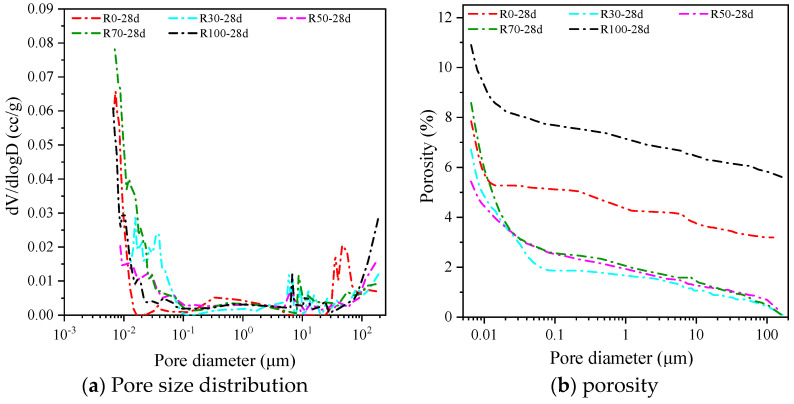
Pore size distribution and porosity of specimens with different replacement rates of RFA at 28 days. (**a**) Distribution of pore size; (**b**) Porosity distribution of different pore sizes.

The pore size distribution and porosity of the AARAC samples cured at 28 days were measured using the MIP method shown in Figure 10. The pore size distribution of the alkali-activated slag recycled concrete is depicted in Table 5.

The porosity and pore size distribution have a significant influence on the compressive strength of materials. According to previous studies [80], the pores were categorized into harmless pores (≤20 nm), smaller harmful pores (20 nm~50 nm), harmful pores (50 nm~200 nm), and more harmful pores (≥200 nm). The results of the pore size distribution and porosity of each group are listed in Table 5. The porosity showed a decreasing and then increasing trend with the increase in the RFA replacement rate. When the content of RFA was 50%, the porosity was the lowest, 5.43%, and when the content of RFA was 100%, the porosity reached the maximum, 10.90%. These were 30.82% lower and 38.93% higher than those of the test group without the RFA, respectively. This indicated that the inclusion of the appropriate amount of RFA could improve the densification of the hardened paste, but when the amount is more than 50%, it adversely affects the densification of the hardened paste. However, a content that is greater than 50% would have an unfavorable effect on the compactness of the hardened paste. In terms of the pore size distribution, when the contents of RFA were 0, 50%, and 100%, the proportion of harmful pores and poly-harmful pores in all pores were 65.92%, 52.12%, and 72.10%, which led to the low compressive strength of the hardened paste. The proportions of harmful pores and poly-harmful pores in the experimental group with an RFA content of 70% were small, so the compressive strength of this group was relatively high, which was consistent with the results of the compressive strength in Section 3.2.1.

### 3.6. SEM Characterization

**Figure 11:**
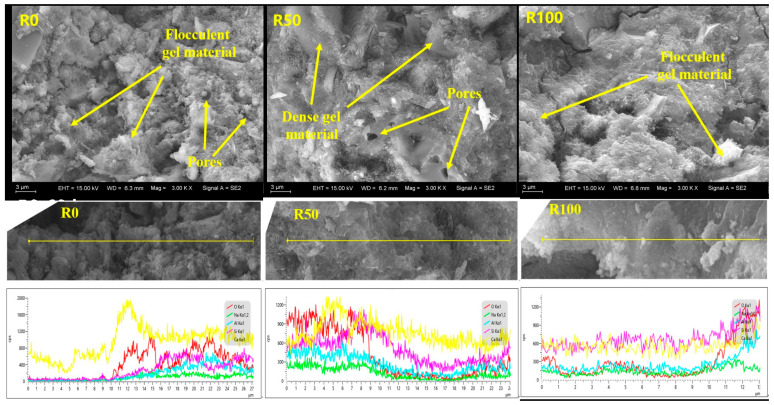
SEM images and line scans of AARAC for different curing ages.

Figure 11 shows the SEM and EDS images of specimens with 0, 50, and 100 wt% RFA at a curing age of 28 days. The regions of the samples containing the reaction products were selected and observed under SEM, and the chemical compositions of the different products were analyzed using EDS. When the curing age was prolonged, the reaction of the AARAC progressed further, the structure of the reaction products became denser, and fewer pores were observed. From the line scanning results, it can be seen that, at a curing age of 28 days, there was a higher content of calcium, which was mainly due to the reaction between SiO_3_^2−^ from the alkali activator and the Ca^2+^ dissolved in the slag. In addition, additional silicate materials in the system reacted with the Ca^2+^ dissolved in the slag to form dense C-A-S-H reaction products [81].

## 4. Conclusions

This study mainly analyzed the effect of the replacement rate of recycled fine aggregate (RFA) on the mechanical properties and drying shrinkage of alkali-activated recycled aggregate concrete (AARAC), and our conclusions can be summarized as follows:

(1) As the replacement rate of RFA was increased, the flowability showed a rapidly decreasing trend. When the replacement rate of RFA was 100 wt%, compared with the test group without the RFA, the flowability was decreased by 35.3%. Adding a certain amount of RFA could improve the mechanical properties of AARAC. A high compressive strength of 65.3 MPa was obtained at an age of 28 days when the replacement rate of the RFA was 70 wt%, which provides the possibility of using high replacement rates for recycled aggregate.

(2) With the prolongation of the curing time, the splitting tensile strength, flexural strength, tension-to-compression ratio, and flexure-to-compression ratio increased and then decreased. When the RFA content was 100 wt%, the splitting tensile strength reached the maximum value of 4.5 MPa after 7 days. With the increase in the replacement rate of the RFA, the flexural strength of the concrete also increased accordingly but remained lower than that of the experimental group without the RFA. With RFA contents of 0 and 100 wt%, the flexural strength values at a curing age of 28 days were 5.5 MPa and 5.11 MPa, respectively. The flexural strength was only decreased by 7.1%, which indicated that the addition of the RFA had minimal impact on the flexural strength of the AARAC. At a curing age of 7 days, the tension-to-compression and flexure-to-compression ratios were both higher (except for R100) than those of the other ages, indicating good levels of ductility and toughness.

(3) The addition of the RFA increased the drying shrinkage of the alkali-activated recycled concrete. At a curing age of 120 days, the experimental group without the RFA had the lowest shrinkage rate of 1049.86 μm. The test group with the addition of 70 wt% RFA had the highest shrinkage rate of 1408.37 μm, and its drying shrinkage rate increased by 34.15%.

(4) With the increase in the curing age, the reaction of the AARAC proceeded further, and the structure of the reaction products became denser. However, the proportion of harmful and multi-harmful pores increased to varying degrees. At the same curing age, the RFA reduced the porosity when the replacement rate was relatively low. Compared with the samples without the RFA, the porosity of the samples with 30 wt% and 50 wt% RFA decreased by 14.49% and 30.83% at 28 days.

Our experimental results showed that the RFA-based AARAC had acceptable mechanical and durability properties. It is feasible to use recycled fine aggregates in concrete production. The combined use of AARAC and RFA can significantly reduce our consumption of natural resources. Meanwhile, RFA-based AARAC could greatly reduce the burden caused by the manufacturing of cement. Applications of this kind of environmentally friendly material are considered to be in line with sustainable development strategies.

## Figures and Tables

**Table 1:** Chemical composition (wt%) of FA and GGBS.

Oxide	SiO_2_	Al_2_O_3_	CaO	Fe_2_O_3_	MgO	Na_2_O	K_2_O	TiO_2_	LOI
FA	45.60	35.45	5.78	6.09	0.67	0.44	2.85	1.88	1.24
GGBS	21.75	16.01	50.40	0.61	6.37	0.30	0.25	0.98	3.33

**Table 2:** Mechanical properties of aggregates.

Property	Natural Fine Aggregates (NFAs)	Recycled Fine Aggregates (RFAs)	Recycled Coarse Aggregates (RCAs)
Maximum Size	4.75 mm	4.75 mm	31.5 mm
Specific Gravity	2587 kg/m^3^	2467 kg/m^3^	2565 kg/m^3^
Aggregate Crushing Value	-	-	14.9
Water Absorption	4.86%	8.18%	3.40%
Bulk Density (Dry Loose State)	1587 kg/m^3^	1460 kg/m^3^	1352 kg/m^3^
Fineness Modulus	2.94	3.05	-

Note: The modulus of fineness is a dimensionless index that characterizes the degree of coarseness and fineness of sand, i.e., the average grain size of sand. The fineness modulus of medium sand is 3.0–2.3.

**Table 3:** Mix proportions of AARAC.

Mix-ID	Concrete Material Usage (Kg/m^3^)								
	GGBS	FA	NFA	RFA	RCA	Sodium Silicate Solution	NaOH	Liquid–Binder Ratio	Water
R0	450	50	630.29	0	945.44	246.15	23.53	0.65	54.59
R30	450	50	441.20	189.09	945.44	246.15	23.53	0.65	54.59
R50	450	50	315.15	315.15	945.44	246.15	23.53	0.65	54.59
R70	450	50	189.09	441.20	945.44	246.15	23.53	0.65	54.59
R100	450	50	0	630.29	945.44	246.15	23.53	0.65	54.59

Note: In order to ensure a constant liquid–binder ratio, the difference in water absorption between NFA and RFA was taken into account in the design of the proportions.

**Table 4:** Test program.

NaOH Concentration (mol/L)	Solution Volume (mL)	RFA (g)	Immersion Time				
			10 min	3 h	12 h	24 h	48 h
0	40	10	A_1_	A_2_	A_3_	A_4_	A_5_
5	40	10	B_1_	B_2_	B_3_	B_4_	B_5_

**Table 5:** Pore size distribution of alkali-activated slag recycled concrete.

Specimens	≤20 nm (%)	20 nm~50 nm (%)	50 nm~200 nm (%)	≥200 nm (%)	Porosity (%)
R0-28d	29.77	1.30	4.22	61.70	7.85
R30-28d	41.06	22.96	3.34	27.60	6.71
R50-28d	32.99	14.28	9.85	42.27	5.43
R70-28d	55.13	10.86	4.59	28.45	8.58
R100-28d	23.63	3.49	3.44	68.66	10.90

## Data Availability

Data are contained within the article.
